# Republished study: long-term toxicity of a Roundup herbicide and a Roundup-tolerant genetically modified maize

**DOI:** 10.1186/s12302-014-0014-5

**Published:** 2014-06-24

**Authors:** Gilles-Eric Séralini, Emilie Clair, Robin Mesnage, Steeve Gress, Nicolas Defarge, Manuela Malatesta, Didier Hennequin, Joël Spiroux de Vendômois

**Affiliations:** 1 Institute of Biology, EA 2608 and CRIIGEN and Risk Pole, MRSH-CNRS, Esplanade de la Paix, University of Caen, Caen, Cedex 14032 France; 2 Department of Neurological, Neuropsychological, Morphological and Motor Sciences, University of Verona, Verona, 37134 Italy; 3 Risk Pole, MRSH-CNRS, Esplanade de la Paix, University of Caen, Caen, Cedex 14032 France

**Keywords:** Genetically modified, GMO, Roundup, NK603, Rat, Glyphosate-based herbicides, Endocrine disruption

## Abstract

**Background:**

The health effects of a Roundup-tolerant NK603 genetically modified (GM) maize (from 11% in the diet), cultivated with or without Roundup application and Roundup alone (from 0.1 ppb of the full pesticide containing glyphosate and adjuvants) in drinking water, were evaluated for 2 years in rats. This study constitutes a follow-up investigation of a 90-day feeding study conducted by Monsanto in order to obtain commercial release of this GMO, employing the same rat strain and analyzing biochemical parameters on the same number of animals per group as our investigation. Our research represents the first chronic study on these substances, in which all observations including tumors are reported chronologically. Thus, it was not designed as a carcinogenicity study. We report the major findings with 34 organs observed and 56 parameters analyzed at 11 time points for most organs.

**Results:**

Biochemical analyses confirmed very significant chronic kidney deficiencies, for all treatments and both sexes; 76% of the altered parameters were kidney-related. In treated males, liver congestions and necrosis were 2.5 to 5.5 times higher. Marked and severe nephropathies were also generally 1.3 to 2.3 times greater. In females, all treatment groups showed a two- to threefold increase in mortality, and deaths were earlier. This difference was also evident in three male groups fed with GM maize. All results were hormone- and sex-dependent, and the pathological profiles were comparable. Females developed large mammary tumors more frequently and before controls; the pituitary was the second most disabled organ; the sex hormonal balance was modified by consumption of GM maize and Roundup treatments. Males presented up to four times more large palpable tumors starting 600 days earlier than in the control group, in which only one tumor was noted. These results may be explained by not only the non-linear endocrine-disrupting effects of Roundup but also by the overexpression of the EPSPS transgene or other mutational effects in the GM maize and their metabolic consequences.

**Conclusion:**

Our findings imply that long-term (2 year) feeding trials need to be conducted to thoroughly evaluate the safety of GM foods and pesticides in their full commercial formulations.

*Empirical natural and social sciences produce knowledge (in German: Wissenschaften schaffen Wissen) which should describe and explain past and present phenomena and estimate their future development. To this end quantitative methods are used. Progress in science needs controversial debates aiming at the best methods as basis for objective, reliable and valid results approximating what could be the truth. Such methodological competition is the energy needed for scientific progress. In this sense, ESEU aims to enable rational discussions dealing with the article from G.-E. Séralini et al. (Food Chem. Toxicol. 2012, 50:4221–4231) by re-publishing it. By doing so, any kind of appraisal of the paper’s content should not be connoted. The only aim is to enable scientific transparency and, based on this, a discussion which does not hide but aims to focus methodological controversies. -Winfried Schröder, Editor of the Thematic Series “Implications for GMO-cultivation and monitoring” in Environmental Sciences Europe.*

## Background

There is an ongoing international debate as to the necessary length of mammalian toxicity studies, including metabolic analyses, in relation to the consumption of genetically modified (GM) plants [1]. Currently, no regulatory authority requires mandatory chronic animal feeding studies to be performed for edible genetically modified organisms (GMOs), or even short-term studies with blood analyses for the full commercial formulations of pesticides as sold and used, but only for the declared active principle alone. However, several 90-day rat feeding trials have been conducted by the agricultural biotechnology industry. These investigations mostly concern GM soy and maize that are engineered either to be herbicide-tolerant (to Roundup (R) in 80% of cases), or to produce a modified Bt toxin insecticide, or both. As a result, these GM crops contain new pesticide residues for which new maximum residue levels (MRL) have been established in some countries.

Though the petitioners conclude in general that no major physiological changes is attributable to the consumption of the GMO in subchronic toxicity studies [2–5], significant disturbances have been found and may be interpreted differently [6,7]. A detailed analysis of the data in the subchronic toxicity studies [2–5] has revealed statistically significant alterations in kidney and liver function that may constitute signs of the early onset of chronic toxicity. This may be explained at least in part by pesticide residues in the GM feed [6,7]. Indeed, it has been demonstrated that R concentrations in the range of 10^3^ times below the MRL can induce endocrine disturbances in human cells [8] and toxic effects thereafter [9]. This may explain toxic effects seen in experiments in rats *in vivo* [10] as well as in farm animals [11]. After several months of consumption of an R-tolerant soy, the liver and pancreas of mice were affected, as highlighted by disturbances in sub-nuclear structure [12–14]. Furthermore, this toxic effect was reproduced by the application of R herbicide directly to hepatocytes in culture [15].

More recently, long-term and multi-generational animal feeding trials have been performed, with some possibly providing evidence of safety, while others conclude on the necessity of further investigation because of metabolic modifications [16]. However, in contrast with the study we report here, none of these previous investigations have included a detailed follow-up of the animals, including multiple (up to 11) blood and urine sampling over 2 years, and none has investigated either the GM NK603 R-tolerant maize or Roundup.

Furthermore, evaluation of long-term toxicity of herbicides is generally performed on mammalian physiology employing only their active principle, rather than the complete formulations as used in agriculture. This was the case for glyphosate (G) [17], the declared active chemical constituent of R. It is important to note that G is only able to efficiently penetrate target plant organisms with the help of adjuvants present in the various commercially used R formulations [18]. Even if G has shown to interact directly with the active site of aromatase at high levels [19], at low contaminating levels, adjuvants may be better candidates than G to explain the toxicity or endocrine disruptive side effects of R on human cells [8,20] and also *in vivo* for acute toxicity [21]. In this regard, it is noteworthy that the far greater toxicity of full agricultural formulations compared to declared supposed active principles alone has recently been demonstrated also for six other major pesticides tested *in vitro* [22]. When G residues are found in tap water, food, or feed, they arise from the total herbicide formulation although little data is available as to the levels of the R adjuvants in either the environment or food chain. Indeed, adjuvants are rarely monitored in the environment, but some widely used adjuvants (surfactants) such as nonylphenol ethoxylates, another ethoxylated surfactant like POEA present in R, are widely found in rivers in England and are linked with disruption of wildlife sexual reproduction [23]. Adjuvants are found in groundwater [24]. The half-life of POEA (21 to 42 days) is even longer than for G (7 to 14 days) in aquatic environments [25]. As a result, the necessity of studying the potential toxic effects of total chemical mixtures rather than single components has been strongly emphasized [26–28]. On this basis, the regular measurement of only G or other supposed active ingredients of pesticides in the environment constitute at best markers of full formulation residues. Thus, in the study of health effects, exposure to the diluted whole formulation may be more representative of environmental pollution than exposure to G alone.

With a view to address this lack of information, we performed a 2-year detailed rat feeding study. Our study was designed as a chronic toxicity study and as a direct follow-up to a previous investigation on the same NK603 GM maize conducted by the developer company, Monsanto [3]. A detailed critical analysis of the raw data of this subchronic 90-day rat feeding study revealed statistically significant differences in multiple organ function parameters, especially pertaining to the liver and kidneys, between the GM and non-GM maize-fed group [3,7]. However, Monsanto's authors dismissed the findings as not ‘biologically meaningful’ [3], as was also the case with another GM corn [29]. The European Food Safety Authority (EFSA) accepted Monsanto's interpretation on NK603 maize [30], like in all other cases.

Our study is the first and to date the only attempt to follow up Monsanto's investigation and to determine whether the differences found in the NK603 GM maize-fed rats, especially with respect to liver and kidney function, were not biologically meaningful, as claimed, or whether they developed into serious diseases over an extended period of time.

The Monsanto authors adapted Guideline 408 of the Organization for Economic Co-operation and Development (OECD) for their experimental design [3]. Our study design was based on that of the Monsanto investigation in order to make the two experiments comparable, but we extended the period of observation from Monsanto's 90 days to 2 years. We also used three doses of GMOs (instead of Monsanto's two) and Roundup to determine treatment dose response, including any possible non-linear as well as linear effects. This allowed us to follow in detail the potential health effects and their possible origins due to the direct or indirect consequences of the genetic modification itself in the NK603 GM maize, or due to the R herbicide formulation used on the GM maize (and not G alone), or both. Because of recent reviews on GM foods indicating no specific risk of cancer [2,16], but indicating signs of hepatorenal dysfunction within 3 months [1,7], we had no reason to adopt a carcinogenesis protocol using 50 rats per group. However, we prolonged to 2 years the biochemical and hematological measurements and measurements of disease status, as allowed, for example, in OECD protocols 453 (combined chronic toxicity and carcinogenicity) and 452 (chronic toxicity). Both OECD 452 and 453 specify 20 rats per sex per group but require only 50% (ten per sex per group, the same number that we used in total) to be analyzed for biochemical and hematological parameters. Thus, these protocols yield data from the same number of rats as our experiment. This remains the highest number of rats regularly measured in a standard GM diet study, as well as for a full formulated pesticide at very low environmentally relevant levels.

We used the Sprague-Dawley strain of rat, as recommended for chronic toxicology tests by the National Toxicology Program in the USA [31], and as used by Monsanto in its 90-day study [3]. This choice is also consistent with the recommendation of the OECD that for a chronic toxicity test, rats of the same strain should be used as in studies on the same substance but of shorter duration [32]. We then also tested for the first time three doses (rather than the two usually employed in 90-day protocols) of the R-tolerant NK603 GM maize alone, the GM maize treated with R, and R alone at very low environmentally relevant doses, starting below the range of levels permitted by regulatory authorities in drinking water and in GM feed.

Overall, our study is the first in-depth life-long toxicology study on the full commercial Roundup formulation and NK603 GM maize, with observations on 34 organs and measurement of 56 parameters analyzed at 11 time points for most organs, and utilizing 3 doses. We report here the major toxicological findings on multiple organ systems. As there was no evidence in the literature on GM food safety evaluation to indicate anything to the contrary, this initial investigation was designed as a full chronic toxicity and not a carcinogenicity study. Thus, we monitored in details chronologically all behavioral and anatomical abnormalities including tumors. A full carcinogenicity study, which usually focuses only on observing incidence and type of cancers (not always all tumors), would be a rational follow-up investigation to a chronic toxicity study in which there is a serious suspicion of carcinogenicity. Such indications had not been previously reported for GM foods.

Our findings show that the differences in multiple organ functional parameters seen from the consumption of NK603 GM maize for 90 days [3,7] escalated over 2 years into severe organ damage in all types of test diets. This included the lowest dose of R administered (0.1 ppb, 50 ng/L G equivalent) of R formulation administered, which is well below permitted MRLs in both the USA (0.7 mg/L) [33] and European Union (100 ng/L) [34]. Surprisingly, there was also a clear trend in increased tumor incidence, especially mammary tumors in female animals, in a number of the treatment groups. Our data highlight the inadequacy of 90-day feeding studies and the need to conduct long-term (2 years) investigations to evaluate the life-long impact of GM food consumption and exposure to complete pesticide formulations.

## Results

### Biochemical analyses of the maize feed

Standard biochemical compositional analysis revealed no particular differences between the different maize types and diets, the GM and non-GM maize being classified as substantially equivalent, except for transgene DNA quantification. For example, there was no difference in total isoflavones. In addition, we also assayed for other specific compounds, which are not always requested for establishing substantial equivalence. This analysis revealed a consistent and statistically significant (*p <* 0.01) decrease in certain phenolic acids in treatment diets, namely ferulic and caffeic acids. Ferulic acid was decreased in both GM maize and GM maize + R diets by 16% to 30% in comparison to the control diet (889 ± 107, 735 ± 89, respectively, vs. control 1,057 ± 127 mg/kg) and caffeic acid in the same groups by 21% to 53% (17.5 ± 2.1, 10.3 ± 1.3 vs. control 22.1 ± 2.6 mg/kg).

### Anatomopathological observations and liver parameters

All rats were carefully monitored during the experiment for behavior, appearance, palpable tumors, and infections. At least ten organs per animal were weighed and up to 34 analyzed postmortem, at the macroscopic and/or microscopic levels (Table 1). Due to the large quantity of data collected, it cannot all be shown in one report, but we present here the most important findings. There was no rejection by the animals of the diet with or without GM maize, nor any major difference in body weight (data not shown).

**Table 1:** **Protocol used and comparison to existing assessment and to non-mandatory regulatory tests**

**Treatments and analyses**	**In this work**	**Hammond et al. 2004**	**Regulatory tests**
Animals measured/group/sex	10/10 SD rats (200 rats measured)	10/20 SD rats (200 rats measured/total 400)	At least 10 rodents
Duration in months	24 (chronic)	3 (subchronic, 13 weeks)	3
Doses by treatment	3	2	At least 3
Treatments + controls	GMO NK603, GMO NK603 + Roundup, Roundup, and closest isogenic maize	GMO NK603 + Roundup, closest isogenic maize, and 6 other maize lines non substantially equivalent	GMOs or Chemicals (in standard diet or water)
Animals by cage (same sex)	1 to 2	1	1 or more
Monitoring/week	2	1	1 or more
Organs and tissues studied			For high dose and controls
Organs weighted	10	7	At least 8
Histology/animal	34	17/36	At least 30
Electronic microscopy	Yes	No	No
Feed and water consumptions	Measured	For feed only	At least feed
Behavioral studies (times)	2	1 (no protocol given)	1
Ophthalmology (times)	2	0	2
Blood parameters	31 (11 times for most)	31 (2 times)	At least 25 (at least 2 times)
Plasma sex steroids	Testosterone, estradiol	No	No, except if endocrine effects suspected
Number of blood samples/animal	11, each month (0 to 3) then every 3 months	2, weeks 4 and 13	1, at the end
Urine parameters studied	16	18	7 if performed
Number of urine samples	11	2	Optional, last week
Liver tissue parameters	6	0	0
Roundup residues in tissues	Studied	Not studied	Not mandatory
Microbiology in feces or urine	Yes	Yes	No
Transgene in tissues	Studied	Not studied	Not studied

The protocol used in this work was compared to the regulatory assessment of NK603 maize by the company (Hammond et al. 2004), and to non-mandatory regulatory *in vivo* tests for GMOs, or mandatory for chemicals (OECD 408). Most relevant results are shown in this paper.

**Figure 1:**
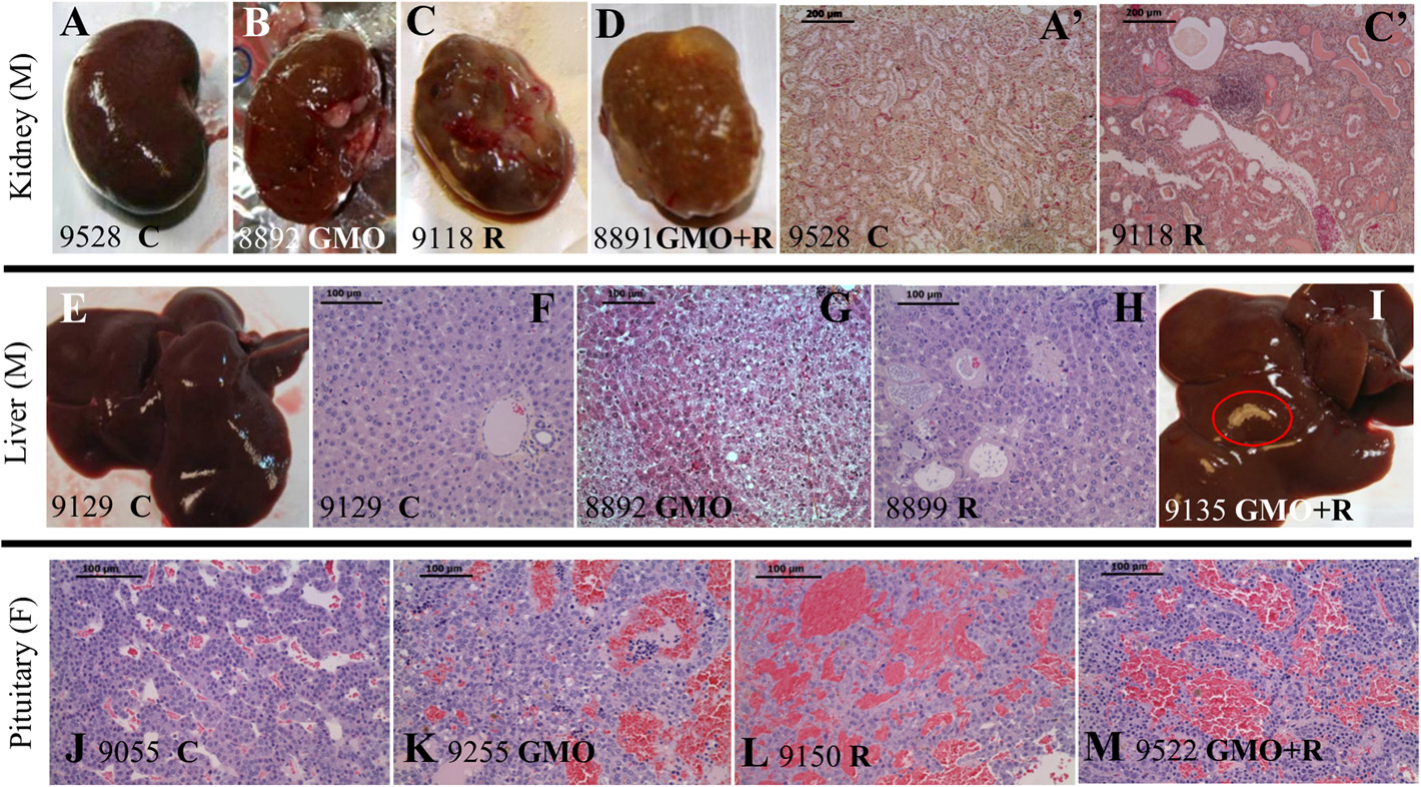
**Anatomopathological observations in rats fed GMO treated or not by Roundup and effects of Roundup alone.** Macroscopic **(A** to **D)** and microscopic **(A'** and **C')** photographs show male left kidneys and livers **(E** to **I)** and female pituitaries **(J** to **M)**, in accordance to Table 2. The number of each animal and its treatment is specified. Macroscopic pale spots **(I)** and microscopic necrotic foci in liver **(G** clear-cell focus, **H** basophilic focus with atypia), and marked or severe chronic progressive nephropathies, are illustrated. In females, pituitary adenomas **(K** to **M)** are shown and compared to control **(J**, rat number and **C** for control). Apostrophes after letters indicate organs from the same rat.

The most affected organs in males were the liver, hepatodigestive tract, and kidneys (Table 2; Figure 1A,B,C,D,E,F,G,H,I). Liver abnormalities such as hepatic congestions and macroscopic and microscopic necrotic foci were 2.5 to 5.5 times more frequent in all treatments than in control groups, where only two rats out of ten were affected with one abnormality each. For instance, there were 5 abnormalities in total in the GMO 11% group (2.5 times higher than controls) and 11 in the GMO 22% group (5.5 times greater). In addition, by the end of the experiment, Gamma GT hepatic activity was increased, particularly in the GMO + R groups (up to 5.4 times higher), this probably being reflective of liver dysfunction. Furthermore, cytochrome P450 activity generally increased in the presence of R (either in drinking water or in the GM maize-containing diet) according to the dose and up to 5.7 times greater at the highest dose.

**Table 2:** **Summary of the most frequent anatomical pathologies observed**

**Organs and associated pathologies**	**Controls**	**GMO 11%**	**GMO 22%**	**GMO 33%**	**R (A)**	**R (B)**	**R (C)**	**GMO 11% + R**	**GMO 22% + R**	**GMO 33% + R**
Males										
Kidneys, CPN	3 (3)	4 (4)	5 (5)	7 (7)	6 (6)	5 (5)	3 (3)	5 (5)	4 (4)	4 (4)
Liver	2 (2)	5 (4)	11 (7)	8 (6)	11 (5)	9 (7)	6 (5)	5 (4)	7 (4)	6 (5)
Hepatodigestive tract	6 (5)	10 (6)	13 (7)	9 (6)	23 (9)	16 (8)	9 (5)	9 (6)	13 (6)	11 (7)
Females										
Pituitary	9 (6)	23 (9)	20 (8)	8 (5)	22 (8)	16 (7)	13 (7)	19 (9)	9 (4)	19 (7)
Mammary glands	10 (5)	22 (8)	10 (7)	16 (8)	26(10)	20(10)	18 (9)	17 (8)	16 (8)	15 (9)
Mammary tumors	8 (5)	15 (7)	10 (7)	15 (8)	20 (9)	16(10)	12 (9)	10 (6)	11 (7)	13 (9)

After the number of pathological abnormalities, the number of rats affected out of the initial ten is indicated in parentheses. Only marked or severe chronic progressive nephropathies (CPN) are listed in male animals, excluding two nephroblastomas in groups consuming GMO 11% and GMO 22% + Roundup. Hepatodigestive pathological signs in males concern the liver, stomach, and small intestine (duodenum, ileum, or jejunum). Pathological signs in liver are mostly congestions, macroscopic spots, and microscopic necrotic foci. In females, pituitary dysfunctions include adenomas, hyperplasias, and hypertrophies. Mammary fibroadenomas and adenocarcinomas are the major tumors detected; galactoceles and hyperplasias with atypia were also found and added to the pathological signs in mammary glands.

**Figure 2:**
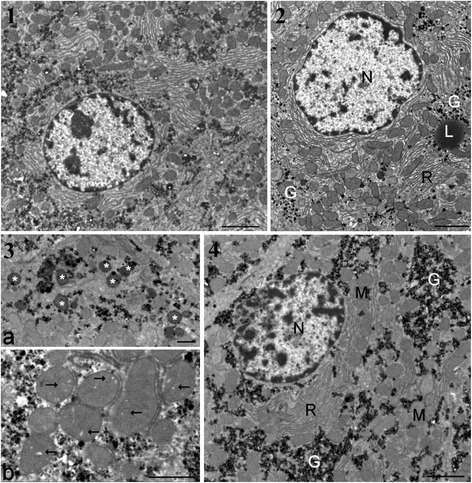
**Ultrastructure of hepatocytes in male rats from groups presenting the greatest degree of liver pathology. (1)** Typical control rat hepatocyte (bar 2 μm except in 4). **(2)** Effects with Roundup at the lowest dose. Glycogen (G) is dispersed in the cytoplasm. L, lipid droplet; N, nucleus; R, rough endoplasmic reticulum. **(3)** Details of treatment effects with 22% dietary GMO (bar 1 μm). a, cluster of residual bodies (asterisks); b, mitochondria show many enlarged cristae (arrows). **(4)** Hepatocytes of animal fed GM maize (GMO) at 22% of total diet. Large lakes of glycogen occur in the cytoplasm. M, mitochondria.

Transmission electron microscopic observations of liver samples confirmed changes for all treated groups in relation to glycogen dispersion or appearance in lakes, increase of residual bodies and enlargement of cristae in mitochondria (Figure 2, panels 2 to 4). The GM maize-fed groups either with or without R application showed a higher heterochromatin content and decreased nucleolar dense fibrillar components, implying a reduced level of mRNA and rRNA transcription. In the GMO + R group (at the highest dose), the smooth endoplasmic reticulum was drastically increased and nucleoli decreased in size, becoming more compact. In the R alone treatment groups, similar trends were observed, with a partial resumption of nucleolar activity at the highest dose.

Degenerating kidneys with turgid inflammatory areas demonstrated the increased incidence of marked and severe chronic progressive nephropathies, which were up to two fold higher in the 33% GM maize or lowest dose R treatment groups (Table 2; Figure 1, first line).

### Biochemical analyses of blood and urine samples

**Figure 3:**
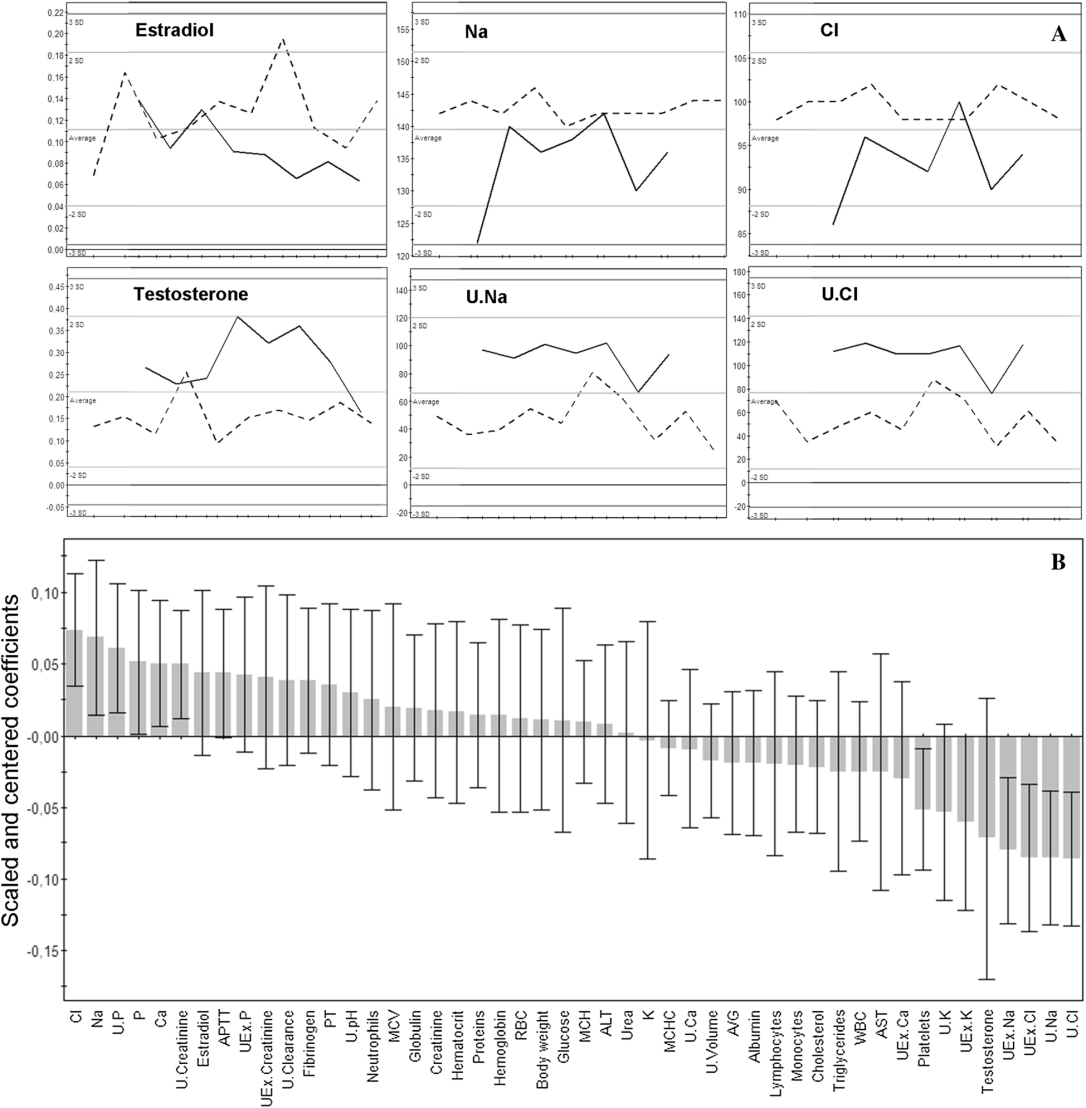
**Orthogonal partial least squares-discriminant analysis (OPLS-DA) for biochemical data (females fed 33% GMO versus controls). (A)** First, detailed examples of significant discriminant variables distribution between females fed 33% GMO (bold line) and controls (dotted line). On *X* axis, animals; on *Y* axis, serum or urine biochemical values for Na, Cl, estradiol, testosterone. **(B)** Wider view of OPLS-DA regression coefficients for predictive component, with jack-knifed confidence intervals at 99% confidence level, indicating discriminant parameters versus controls at month 15. U, urinary; UEx, excreted in urine during 24 h; APPT, activated partial thromboplastin time; MCV, mean corpuscular volume; PT, prothrombine time; RBC, red blood cells; ALT, alanine aminotransferase; MCHC, mean corpuscular hemoglobin concentration; A/G, albumin/globulin ratio; WBC, white blood cells; AST, aspartate aminotransferase. Profiles evidence kidney ion leakages and sex hormonal imbalance versus controls.

Biochemical measurements of blood and urine were focused on samples taken at the 15th month time point, as this was the last sampling time when most animals were still alive (in treated groups 90% males, 94% females, and 100% controls). Statistical analysis of results employed OPLS-DA 2-class models built between each treated group per sex and controls. Only models with an explained variance R^2^(Y) ≥ 80%, and a cross-validated predictive ability Q^2^(Y) ≥ 60%, were used for selection of the discriminant variables (Figure 3), when their regression coefficients were significant at a 99% confidence level. Thus, in treated females, kidney failures appeared at the biochemical level (82% of the total disrupted parameters). Levels of Na and Cl or urea increased in urine with a concomitant decrease of the same ions in serum, as did the levels of P, K, and Ca. Creatinine and creatinine clearance decreased in urine for all treatment groups in comparison to female controls (Table 3). In GM maize-treated males (with or without R), 87% of discriminant variables were kidney-related, but the disrupted profiles were less obvious because of advanced chronic nephropathies and deaths. In summary, for all treatments and both sexes, 76% of the discriminant variables versus controls were kidney-related.

**Table 3:** **Percentage variation of parameters indicating kidney failures of female animals**

**Discriminant variables**		**GMO 11%**	**GMO 22%**	**GMO 33%**	**GMO 11% + R**	**GMO 22% + R**	**GMO 33% + R**	**R (A)**	**R (B)**	**R (C)**
Gonadal hormones	Estradiol	5	−2	−25	8	−1	2	−26	*−*73^a^	39
	Testosterone	56^a^	17	81	5	−9	27	97^a^	*−*72^a^	10
Serum decrease or increase	Na	−1	*−*4^a^	*−*6^a^	2	1	1	*−7*	0	−3
	Cl	−5	*−7*	*−*6^a^	−1	−2	−2	*−*8^a^	−1	−4
	P	−17	*−*18^a^	*−*20^a^	−6	−11	−13	*−*32^a^	−9	−13
	K	2	−4	0	4	5	10	−4	8	*−*5^a^
	Ca	2^a^	−2	*−*5^a^	4	3	3	−6	3	*−*6^a^
Urinary increase	Urea	15	12	−1	12	18^a^	15	0	13	32^a^
	Na	52	−2	95^a^	25	33	30	62^a^	65	91^a^
	Na ex	50	24	125^a^	24	50	68	108^a^	51	7
	Cl	46	5	101^a^	14	35	28	67^a^	56	94^a^
	Cl ex	51	31	138^a^	20	63	70	121^a^	48	13
Urinary decrease	Clearance	*−*20^a^	*−*20^a^	−19	−4	−11	−20	*−*20^a^	*−*24^a^	*−*40^a^
	Creatinine	−19	−37	*−*36^a^	−5	*−*32^a^	*−*37^a^	−43	−23	−1
	Creatinine ex	−18	*−*17^a^	−21	−5	−11	*−*19^a^	*−*21^a^	*−*22^a^	*−*39^a^

OPLS-DA was performed on 48 variables at month 15. Here, we show mean differences (%) of variables (^a^discriminant at 99% confidence level) indicating kidney parameters of female animals, together with sex hormones. Male kidney pathologies are already illustrated in Figure 1.

Furthermore, in females (Table 3), the androgen/estrogen balance in serum was modified by GM maize and R treatments (at least 95% confidence level, Figure 3). For male animals at the highest R treatment dose, levels of estrogens were more than doubled.

### Tumor incidence

**Figure 4:**
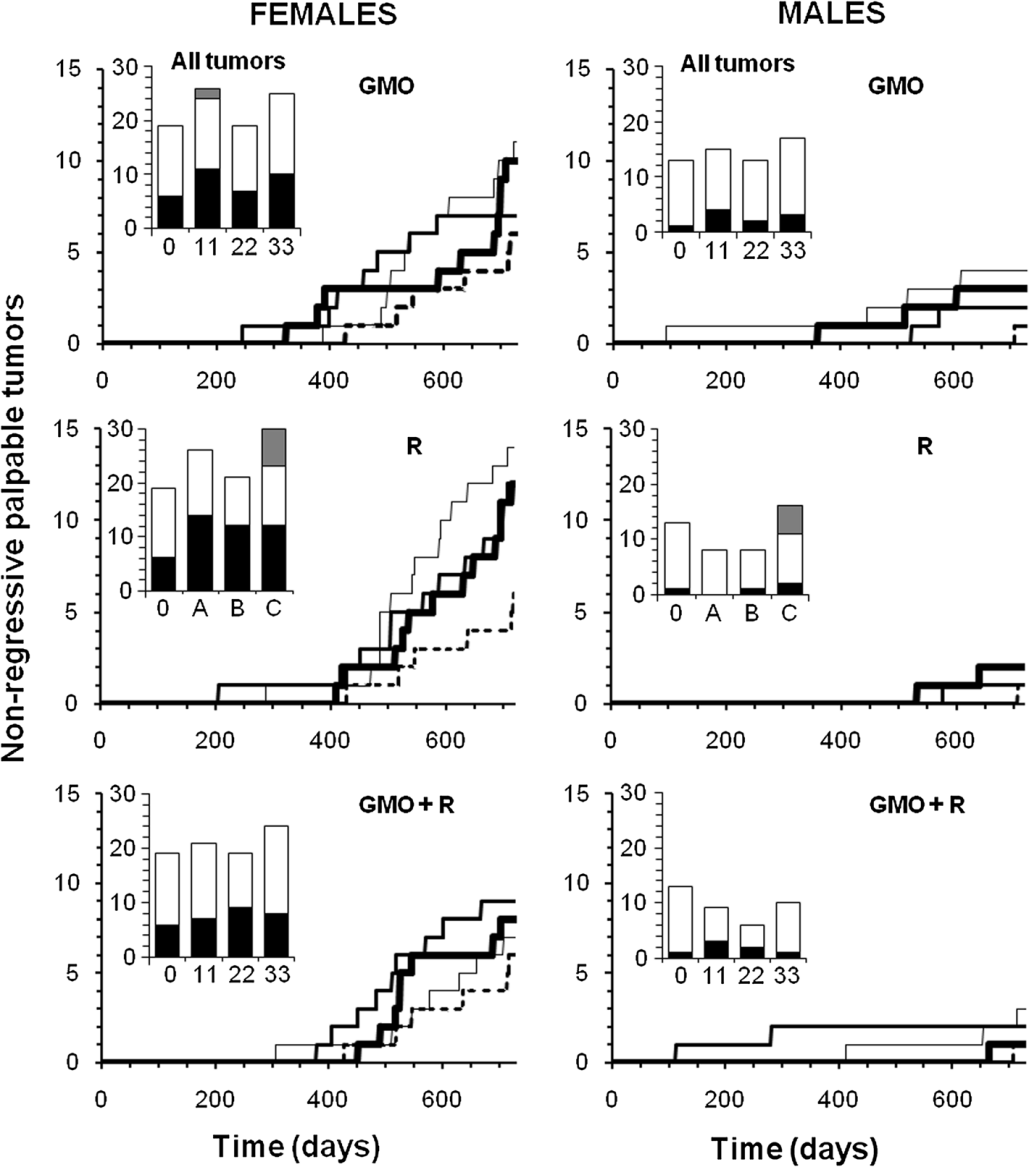
**Largest non-regressive tumors in rats fed GMO treated or not by Roundup and effects of Roundup alone.** Rats were fed with NK603 GM maize (with or without application of Roundup) at three different doses (11%, 22%, and 33% in their diet; thin, medium, and bold lines, respectively) compared to the substantially equivalent closest isogenic non-GM maize (control, dotted line). Roundup was administered in drinking water at three increasing doses, same symbols, environmental **(A)**, MRL in some agricultural GMOs **(B)**, and half of minimal agricultural levels **(C)**, see ‘Methods’). The largest tumors were palpable during the experiment and numbered from 20 mm in diameter for males and 17.5 mm for females. Above this size, 95% of growths were non-regressive tumors. Summary of all tumors are shown in the bar histograms: black, non-regressive large tumors; white, small internal tumors; grey, metastases.

**Figure 5:**
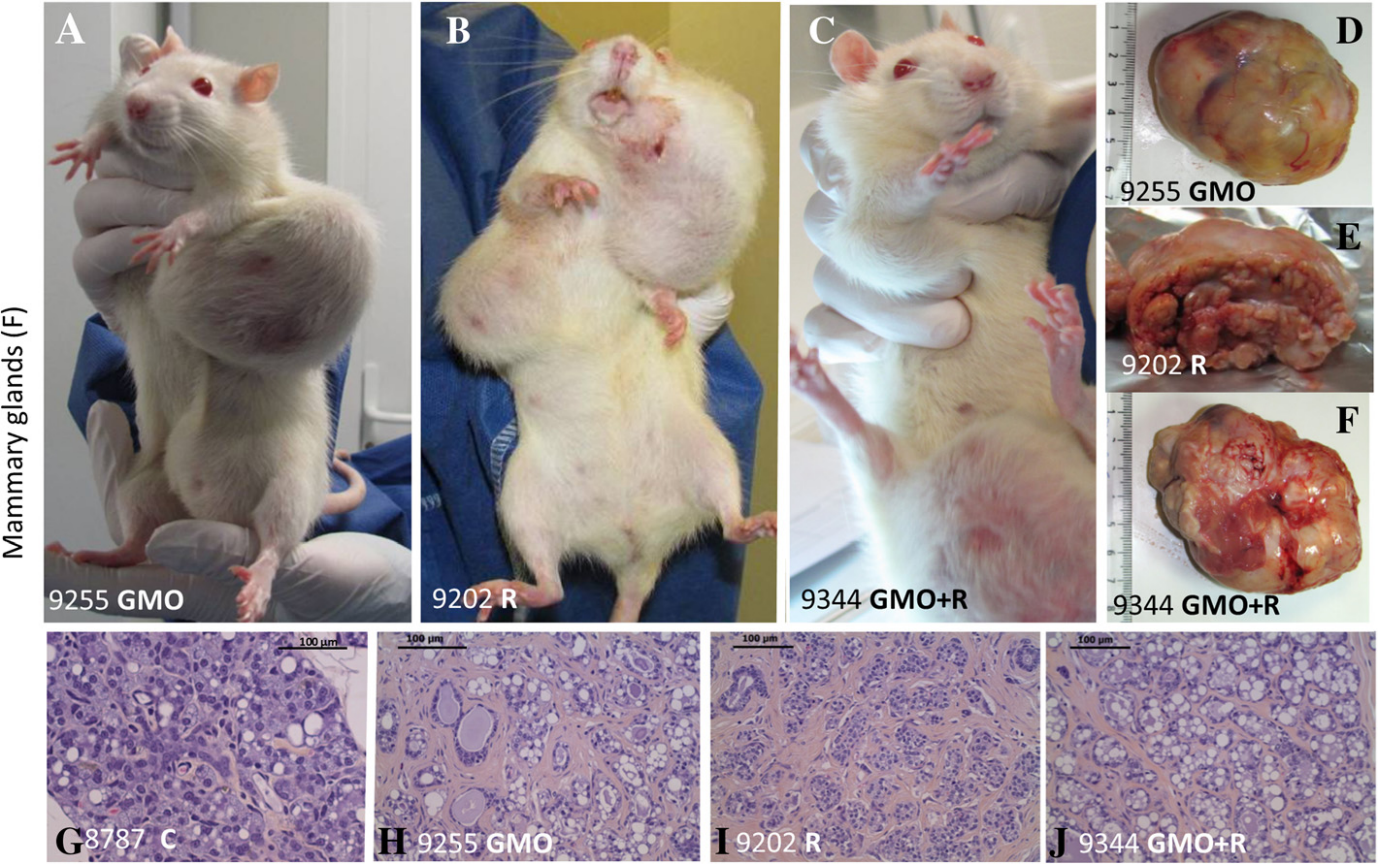
**Examples of female mammary tumors observed.** Mammary tumors are evidenced **(A**, **D**, **H**, representative adenocarcinoma, from the same rat in a GMO group) and in Roundup and GMO + Roundup groups, two representative rats (**B**, **C**, **E**, **F**, **I**, **J** fibroadenomas) are compared to controls. A normal representative rat in controls is not shown, only a minority of them having tumors up to 700 days, in contrast with the majority affected in all treated groups. **(G)** The histological control.

**Figure 6:**
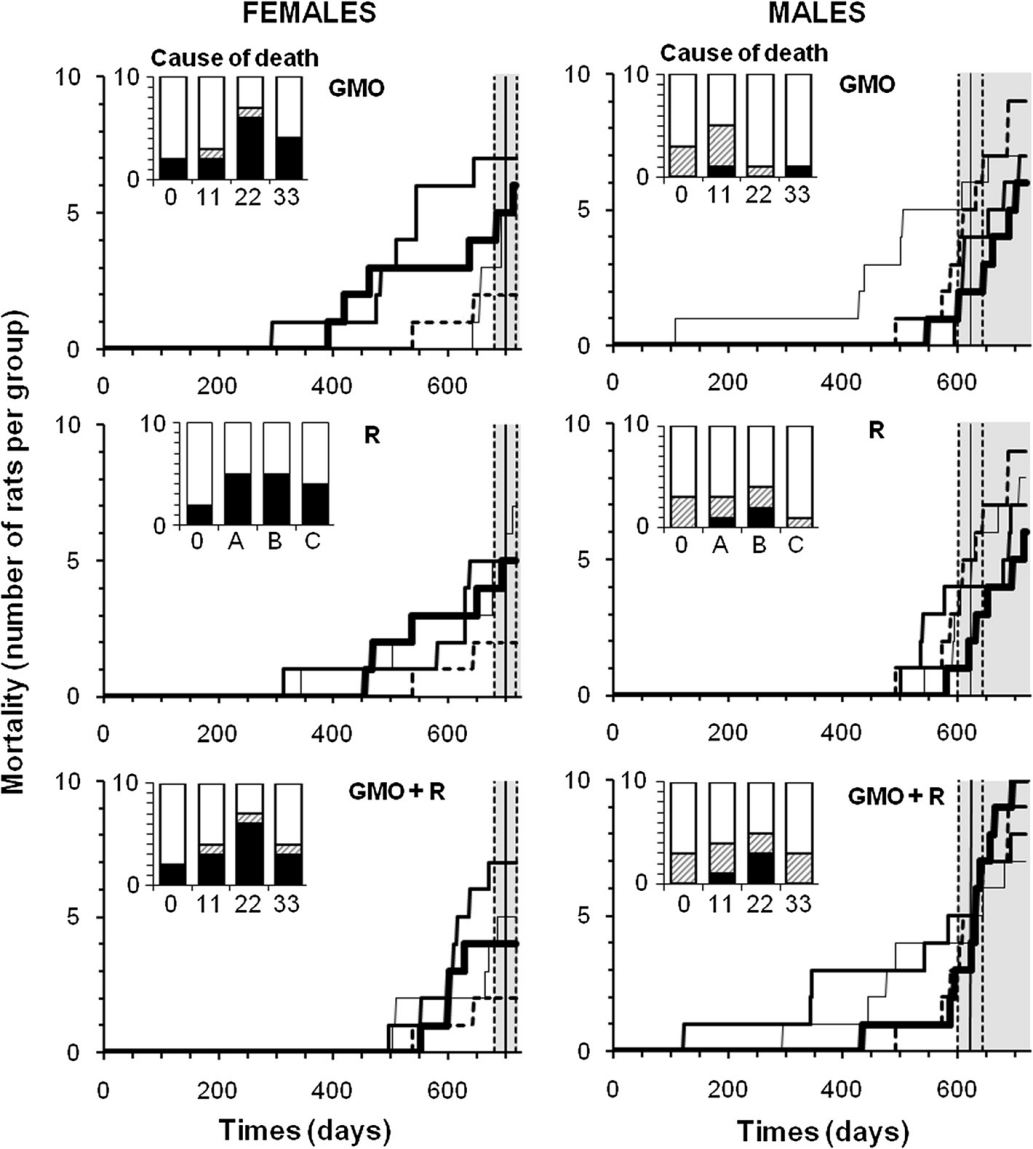
**Mortality of rats fed GMO treated or not with Roundup and effects of Roundup alone.** The symbols of curves and treatments are explained in the caption of Figure 4. Lifespan during the experiment for the control group is represented by the vertical bar ± SEM (grey area). In bar histograms, the causes of mortality before the grey area are detailed in comparison to the controls (0). In black are the necessary euthanasia because of suffering in accordance with ethical rules (tumors over 25% body weight, more than 25% weight loss, hemorrhagic bleeding, etc.); and in hatched areas, spontaneous mortality.

Tumors are reported in line with the requirements of OECD chronic toxicity protocols 452 and 453, which require all ‘lesions’ (which by definition include tumors) to be reported. These findings are summarized in Figure 4. The results are presented in the form of real-time cumulative curves (each step corresponds to an additional tumor in the group). Only the growing largest palpable growths (above a diameter of 17.5 mm in females and 20 mm in males) are presented (for example, see Figure 5A,B,C). These were found to be in 95% of cases non-regressive tumors (Figure 5D,E,F,G,H,I,J) and were not infectious nodules. These arose from time to time; then, most often disappeared and were not different from controls after bacterial analyses. The real tumors were recorded independently of their grade, but dependent on their morbidity, since non-cancerous tumors can be more lethal than those of cancerous nature, due to internal hemorrhaging or compression and obstruction of function of vital organs, or toxins or hormone secretions. These tumors progressively increased in size and number, but not proportionally to the treatment dose, over the course of the experiment (Figure 4). As in the case of rates of mortality (Figure 6), this suggests that a threshold in effect was reached at the lower doses. Tumor numbers were rarely equal but almost always more than in controls for all treated groups, often with a two- to threefold increase for both sexes. Tumors began to reach a large size on average 94 days before controls in treated females and up to 600 days earlier in two male groups fed with GM maize (11 and 22% with or without R).

In female animals, the largest tumors were in total five times more frequent than in males after 2 years, with 93% of these being mammary tumors. Adenomas, fibroadenomas, and carcinomas were deleterious to health due to their very large size (Figure 5A,B,C) rather than the grade of the tumor itself. Large tumor size caused impediments to either breathing or digestion and nutrition because of their thoracic or abdominal location and also resulted in hemorrhaging (Figure 5A,B,C). In addition, one metastatic ovarian cystadenocarcinoma and two skin tumors were identified. Metastases were observed in only two cases; one in a group fed with 11% GM maize and another in the highest dose of R treatment group.

Up to 14 months, no animals in the control groups showed any signs of palpable tumors, whilst 10% to 30% of treated females per group developed tumors, with the exception of one group (33% GMO + R). By the beginning of the 24th month, 50% to 80% of female animals had developed tumors in all treatment groups, with up to three tumors per animal, whereas only 30% of controls were affected. A summary of all mammary tumors at the end of the experiment, independent of size, is presented in Table 2. The same trend was observed in the groups receiving R in their drinking water (Figure 4, R treatment panels). The R treatment groups showed the greatest rates of tumor incidence, with 80% of animals affected (with up to three tumors for one female), in each group. Using a non-parametric multiple comparison analysis, mammary tumor incidence was significantly increased at the lowest dose of R compared to controls (*p* < 0.05, Kruskal-Wallis test with *post hoc* Dunn's test). All females except one (with metastatic ovarian carcinoma) presented in addition mammary hypertrophies and in some cases hyperplasia with atypia (Table 2).

The second most affected organ in females was the pituitary gland, in general around two times more than in controls for most treatments (Table 2; Figure 1J,K,L,M). Again, at this level of examination, adenomas and/or hyperplasias and hypertrophies were noticed. For all R treatment groups, 70% to 80% of animals presented 1.4 to 2.4 times more abnormalities in this organ than controls.

The large palpable tumors in males (in kidney and mostly skin) were by the end of the experimental period on average twice as frequent as in controls, in which only one skin fibroma appeared during the 23rd month. At the end of the experiment, internal non-palpable tumors were added, and their sums were lower in males than in females. They were not significantly different from controls, although slightly increased in females (Figure 4, histogram insets).

### Mortality

The rates of mortality in the various control and treatment groups are shown as raw data in Figure 6. Control male animals survived on average 624 ± 21 days, whilst females lived for 701 ± 20 days during the experiment, plus in each case, a 5-week starting age at reception of animals and a 3-week housing stabilization period. After mean survival time had elapsed, any deaths that occurred were considered to be largely due to aging. Before this period, 30% control males (three in total) and 20% females (only two) died spontaneously, while up to 50% males and 70% females died in some groups on diets containing the GM maize (Figure 6, panels GMO, GMO + R). However, the rate of mortality was not proportional to the treatment dose, reaching a threshold at the lowest (11%) or intermediate (22%) amounts of GM maize in the equilibrated diet, with or without the R application on the crop. It is noteworthy that the first two male rats that died in both GM maize-treated groups had to be euthanized due to Wilms' kidney tumors that had grown by this time to over 25% of body weight. This was approximately a year before the first control animal died. The first female death occurred in the 22% GM maize feeding group and resulted from a mammary fibroadenoma 246 days before the first control female death. The maximum difference in males was five times more deaths occurring by the 17th month in the group consuming 11% GM maize and in females six times greater mortality by the 21st month on the 22% GM maize diet with and without R. In the female cohorts, there were two to three times more deaths in all treated groups compared with controls by the end of the experiment and deaths occurred earlier in general. Females were more sensitive to the presence of R in drinking water than males, as evidenced by a shorter lifespan (Figure 6, panels R). The general causes of death represented in histogram format within each of the panels in Figure 6, are linked mostly to mammary tumors in females and to problems in other organ systems in males.

## Discussion

This report describes the first long-term (2-year) rodent (rat) feeding study investigating possible toxic effects arising from consumption of an R-tolerant GM maize (NK603) and a complete commercial formulation of R herbicide. The aims of this investigation were essentially twofold. First, to evaluate whether the signs of toxicity, especially with respect to liver and kidney functions, seen after 90 days' consumption of a diet containing NK603 R-tolerant GM maize [3,7] escalated into serious ill health or dissipated over an extended period of time. Second, to determine if low doses of full commercial R formulation at permitted levels were still toxic, as indicated by our previous *in vitro* studies [8,9]. The previous toxicity study with NK603 maize employed only this GM crop that had been sprayed with R during cultivation [3]. However, in our study presented here, in addition to extending the treatment period from 90 days to 2 years and in order to better ascertain the source of any ill health observed, we included additional test feeding groups. These consisted of NK603 maize grown without as well as with R application and R alone administered via drinking water. Furthermore, we used three levels of dosing in all cases rather than the two previously used [3], in order to highlight any dose response effects of a given treatment. It is also important to note that our study is the first to conduct blood, urine, and organ analyses from animals treated with the complete agricultural formulation of R and not just G alone, as measured by the manufacturer [35].

Our data show that the signs of liver and kidney toxicity seen at 90 days from the consumption of NK603 GM maize [3,7] do indeed escalate into severe disease over an extended period. Furthermore, similar negative health effects were observed in all treatment groups (NK603 GM maize with or without R application and R alone).

What is also evident from our data is that ill effects were not proportional to the dose of either the NK603 GM maize ± R or R alone. This suggests that the observed disease may result from endocrine disruptive effects, which are known to be non-monotonic. Similar degrees of pathological symptoms occurred from the lowest to the highest doses, suggesting a threshold effect [36]. This corresponds to levels likely to arise from consumption or environmental exposure, such as either 11% GM maize in food, or 50 ng/L G equivalent of R-formulation, a level which can be found in some contaminated drinking tap waters and which falls within authorized limits.

Death in male rats was mostly due to the development of severe hepatorenal insufficiencies, confirming the first signs of toxicity observed in 90-day feeding trials with NK603 GM maize [7]. In females, kidney ion leakage was evident at a biochemical level at month 15, when severe nephropathies were observed in dead male animals at postmortem, at the anatomopathological level. Early signs of toxicity at month 3 in kidney and liver were also observed for 19 edible GM crops containing pesticide residues [1]. It is known that only elderly male rats are sensitive to chronic progressive nephropathies [37]. Therefore, the disturbed kidney functional parameters may have been induced by the reduced levels of phenolic acids in the GM maize feed used in our study, since caffeic and ferulic acids are beneficial to the kidney as they prevent oxidative stress [38,39]. This possibility is consistent with our previous observation that plant extracts containing ferulic and caffeic acids were able to promote detoxification of human embryonic kidney cells after culture in the presence of R [40]. It is thus possible that NK603 GM maize consumption, with its reduced levels of these compounds, may have provoked the early aging of the kidney physiology, similarly to R exposure causing oxidative stress [41]. Disturbances in global patterns of gene expression leading to disease via epigenetic effects cannot be excluded, since it has been demonstrated that numerous pesticides can cause changes in DNA methylation and histone modification, thereby altering chromatin compaction and thus gene expression profiles [42].

Disturbances that we found to occur in the male liver are characteristic of chronic toxicity, confirmed by alterations in biochemical liver and kidney function parameters. The observation that liver function in female animals was less negatively affected may be due to the known protection from oxidative stress conferred by estrogen [43]. Estrogen can induce expression of genes such as superoxide dismutase and glutathione peroxidase via the MAP kinase-NF-kB signaling pathway, thus providing an antioxidant effect [43]. Furthermore, liver enzymes have been clearly demonstrated as sex-specific in their expression patterns, including in a 90-day rat feeding trial of NK603 GM maize [7]. However, in a long-term study, evidence of early liver aging was observed in female mice fed with R-tolerant GM soy [12]. In the present investigation, deeper analysis at an ultrastructural level revealed evidence of impediments in transcription and other defects in cell nuclear structure that were comparable in both sexes and dose-dependent in hepatocytes in all treatments. This is consistent with the well-documented toxic effect of very low dilutions of R on apoptosis, mitochondrial function, and cell membrane degradation, inducing necrosis of hepatocytes, and in other cell lines [8,9,44,45].

The disruptions of at least the estrogen-related pathways and/or enhancement of oxidative stress by all treatments need further confirmation. This can be addressed through the application of transcriptomic, proteomic, and metabolomic methods to analyze the molecular profile of kidneys and livers, as well as the GM NK603 maize [46–48]. Other possible causes of observed pathogenic effects may be due to disturbed gene expression resulting from the transgene insertional, general mutagenic, or metabolic effects [49,50] as has been shown for MON810 GM maize [51,52]. A consequent disruption of general metabolism in the GMO cannot be excluded, which could lead, for example, to the production of other potentially active compounds such as miRNAs [53] or leukotoxin diols [54].

The lifespan of the control group of animals corresponded to the mean for the strain of rat used (Harlan Sprague-Dawley), but as is frequently the case with most mammals, including humans [55], males on average died before females, except for some female treatment groups. All treatments in both sexes enhanced large tumor incidence by two- to threefold in comparison to our controls and also the number of mammary tumors in comparison to the Harlan Sprague-Dawley strain [56] and overall around threefold in comparison to the largest study with 1,329 Sprague-Dawley female rats [57]. This indicates that the use of historical data to compare our tumor numbers is not relevant, first, since we studied the difference with concurrent controls chronologically (and not only at the end of the experiment, as is the case in historical data), and second, since the diets of historical reference animals may have been contaminated with several non-monitored compounds including GMOs and pesticides at levels used in our treatments. In our study, the tumors also developed considerably faster than in controls, even though the majority of tumors were observed after 18 months. The first large detectable tumors occurred at 4 and 7 months into the study in males and females, respectively, further underlining the inadequacy of the standard 90-day feeding trials for evaluating GM crop and food toxicity [1]. Future studies employing larger cohorts of animals providing appropriate statistical power are required to confirm or refute the clear trend in increased tumor incidence and mortality rates seen with some of the treatments tested in this study. As already stated, our study was not designed as a carcinogenicity study that would have required according to OECD the use of 50 rats per sex per group. However, we wish to emphasize that the need for more rats to provide sufficient statistical power may be biased by the presence of contaminants in the diets used in gathering historical control data, increasing artificially the background of tumors, which would inappropriately be called in this case ‘spontaneous’ or due to the genetic strain. For instance, toxic, hormonal disrupting or carcinogenic levels of pesticides, PCBs, plasticizers, dioxins, or heavy metals may contaminate the diets or drinking water used for the establishment of ‘spontaneous’ tumors in historical data [58–62].

In females, induced euthanasia due to suffering and deaths corresponded mostly to the development of large mammary tumors. This was observed independently of the cancer grade but according to impact on morbidity. These appeared to be related to the various treatments when compared to the control groups. These tumors are generally known to be mostly estrogen-dependent [63]. We observed a strikingly marked induction of mammary tumors in groups administered R alone, even at the very lowest dose (50 ng/L G equivalent dilution in adjuvants). At this concentration *in vitro*, G alone is known to induce human breast cancer cell growth via estrogen receptors [64]. In addition, R with adjuvants has been shown to disrupt aromatase, which synthesizes estrogen [19], and to interfere with estrogen and androgen receptors in cells [8]. Furthermore, R appears to be a sex endocrine disruptor *in vivo* in males [10]. Sex steroid levels were also modified in treated rats in our study. These hormone-dependent phenomena are confirmed by enhanced pituitary dysfunction in treated females. An estrogen-modified feedback mechanism may act at this level [65,66]. The similar pathological profiles provoked by the GM maize + R diet may thus be explained at least in part by R residues present in this feed. In this regard, it is noteworthy that the medium dose of the R treatment tested (400 mg/Kg G equivalent) corresponds to acceptable residue levels of this pesticide in some edible GMOs.

Interestingly and perhaps surprisingly, in the groups of animals fed with the NK603 GM maize without R application, similar effects with respect to enhanced tumor incidence and mortality rates were observed. For instance, comparing the 11% GMO-treated female group to the controls, the assumption that the tumors are equally distributed is rejected with a level of significance of 0.54% with the Westlake exceedance test [67]. The classical tests of Kolmogorov-Smirnov (one-sided) and Wilcoxon-Mann-Whitney reach α values of significance, which are respectively of 1.40% and 2.62%.

A possible explanation for this finding is the production of specific compound(s) in the GM feed that are either directly toxic and/or cause the inhibition of pathways, which in turn generates toxic effects. This is despite the fact that the variety of GM maize used in this study was judged by industry and regulators as being substantially equivalent to the corresponding non-GM closest isogenic line [3,30]. As the total chemical composition of the GM maize has not been measured in detail, the use of substantial equivalence as a concept in risk assessment is insufficient to highlight potential unknown toxins and therefore cannot replace long-term animal feeding trials for GMOs.

A cause of the ill effects resulting from NK603 GM maize alone observed in this study could be the fact that it is engineered to overexpress a modified version of the *Agrobacterium tumefaciens* 5-enolpyruvylshikimate-3-phosphate synthase (EPSPS-CP4) [3], which confers R tolerance. The modified EPSPS is not inhibited by G, in contrast to the wild-type enzyme in the crop. This enzyme is known to drive the first step of aromatic amino acid biosynthesis in the plant shikimate pathway. In addition, estrogenic isoflavones and their glycosides are also products of this pathway [68]. A limited compositional analysis showed that these biochemical pathways were not disturbed in the GM maize used in our study. However, our analysis did reveal that the levels of caffeic and ferulic acids in the GM diet, which are also secondary metabolites of the plant shikimate pathway, but not always measured in regulatory tests, were significantly reduced. This may lower their protective effects against carcinogenesis and mammalian tumor formation [69,70]. Moreover, these phenolic acids, and in particular ferulic acid, may modulate estrogen receptors or the estrogenic pathway in mammalian cells [71]. This does not exclude the possibility of the action of other unknown metabolites. This explanation also corresponds to the fact that the observed effects of NK603 GM maize and R were not additive but reached a threshold. This implies that both the NK603 maize and R may cause hormonal disturbances in the same biochemical and physiological pathways.

## Conclusions

In conclusion, the consumption of NK603 GM maize with or without R application or R alone gave similar pathologies in male and female rats fed over a 2-year period. It was previously known that G consumption in water above authorized limits may provoke hepatic and kidney failure [33]. The results of the study presented here clearly indicate that lower levels of complete agricultural G herbicide formulations, at concentrations well below officially set safety limits, can induce severe hormone-dependent mammary, hepatic, and kidney disturbances. Similarly, disruption of biosynthetic pathways that may result from overexpression of the EPSPS transgene in the GM NK603 maize can give rise to comparable pathologies that may be linked to abnormal or unbalanced phenolic acid metabolites or related compounds. Other mutagenic and metabolic effects of the edible GMO cannot be excluded. This will be the subject of future studies, including analyses of transgene, G and other R residue presence in rat tissues. Reproductive and multigenerational studies will also provide novel insight into these problems. This study represents the first detailed documentation of long-term deleterious effects arising from consumption of a GMO, specifically a R-tolerant maize, and of R, the most widely used herbicide worldwide.

Taken together, the significant biochemical disturbances and physiological failures documented in this work reveal the pathological effects of these GMO and R treatments in both sexes, with different amplitudes. They also show that the conclusion of the Monsanto authors [3] that the initial indications of organ toxicity found in their 90-day experiment were not ‘biologically meaningful’ is not justifiable.

We propose that agricultural edible GMOs and complete pesticide formulations must be evaluated thoroughly in long-term studies to measure their potential toxic effects.

## Methods

### Ethics

The experimental protocol was conducted in an animal care unit authorized by the French Ministries of Agriculture and Research (Agreement Number A35-288-1). Animal experiments were performed according to ethical guidelines of animal experimentations (CEE 86/609 regulation), including the necessary observations of all tumors, in line with the requirements for a long-term toxicological study [32], up to a size where euthanasia on ethical grounds was necessary.

Concerning the cultivation of the maize used in this study, no specific permits were required. This is because the maize was grown (MON-00603-6 commonly named NK603) in Canada, where it is authorized for unconfined release into the environment and for use as a livestock feed by the Canadian Food Inspection Agency (Decision Document 2002-35). We confirm that the cultivation did not involve endangered or protected species. The GM maize was authorized for import and consumption into the European Union (CE 258/97 regulation).

### Plants, diets, and chemicals

The varieties of maize used in this study were the DKC 2678 R-tolerant NK603 (Monsanto Corp., USA), and its nearest isogenic non-transgenic control DKC 2675. These two types of maize were grown under similar normal conditions, in the same location, spaced at sufficient distance to avoid cross-contamination. The genetic nature, as well as the purity of the GM seeds and harvested material, was confirmed by qPCR analysis of DNA samples. One field of NK603 was treated with R at 3 L ha^−1^ (WeatherMAX, 540 g/L of G, EPA Reg. 524-537), and another field of NK603 was not treated with R. Corn cobs were harvested when the moisture content was less than 30% and were dried at a temperature below 30°C. From these three cultivations of maize, laboratory rat chow was made based on the standard diet A04 (Safe, France). The dry rat feed was made to contain 11%, 22%, or 33% of GM maize, cultivated either with or without R, or 33% of the non-transgenic control line. The concentrations of the transgene were confirmed in the three doses of each diet by qPCR. All feed formulations consisted of balanced diets, chemically measured as substantially equivalent except for the transgene, with no contaminating pesticides over standard limits. All secondary metabolites cannot be known and measured in the composition. However, we measured isoflavones and phenolic acids including ferulic acid by standard HPLC-UV. All reagents used were of analytical grade. The herbicide diluted in the drinking water was the commercial formulation of R (GT Plus, 450 g/L of G, approval 2020448, Monsanto, Belgium). Herbicide levels were assessed by G measurements in the different dilutions by mass spectrometry.

### Animals and treatments

Virgin albino Sprague-Dawley rats at 5 weeks of age were obtained from Harlan (Gannat, France). All animals were kept in polycarbonate cages (820 cm^2^, Genestil, France) with two animals of the same sex per cage. The litter (Toplit classic, Safe, France) was replaced twice weekly. The animals were maintained at 22 ± 3°C under controlled humidity (45% to 65%) and air purity with a 12 h-light/dark cycle, with free access to food and water. The location of each cage within the experimental room was regularly changed. This 2-year life-long experiment was conducted in a Good Laboratory Practice (GLP) accredited laboratory according to OECD guidelines. After 20 days of acclimatization, 100 male and 100 female animals were randomly assigned on a weight basis into ten equivalent groups. For each sex, one control group had access to plain water and standard diet from the closest isogenic non-transgenic maize control; six groups were fed with 11%, 22%, and 33% of GM NK603 maize either treated or not treated with R. The final three groups were fed with the control diet and had access to water supplemented with respectively 1.1 × 10^−8^% of R (0.1 ppb or 50 ng/L of G, the contaminating level of some regular tap waters), 0.09% of R (400 mg/kg G, US MRL of 400 ppm G in some GM feed), and 0.5% of R (2.25 g/L G, half of the minimal agricultural working dilution). This was changed weekly. Twice-weekly monitoring allowed careful observation and palpation of animals, recording of clinical signs, measurement of any tumors, food and water consumption, and individual body weights.

### Anatomopathology

Animals were sacrificed during the course of the study only if necessary because of suffering according to ethical rules (such as 25% body weight loss, tumors over 25% body weight, hemorrhagic bleeding, or prostration) and at the end of the study by exsanguination under isoflurane anesthesia. In each case, detailed observations and anatomopathology was performed and the following organs were collected: brain, colon, heart, kidneys, liver, lungs, ovaries, spleen, testes, adrenals, epididymis, prostate, thymus, uterus, aorta, bladder, bone, duodenum, esophagus, eyes, ileum, jejunum, lymph nodes, lymphoreticular system, mammary glands, pancreas, parathyroid glands, Peyer's patches, pituitary, salivary glands, sciatic nerve, skin, spinal cord, stomach, thyroid, and trachea. The first 14 organs (at least ten per animal depending on the sex, Table 1) were weighted, plus any tumors that arose. The first nine were divided into two parts and one half was immediately frozen in liquid nitrogen/carbonic ice. The remaining parts including other organs were rinsed in PBS and stored in 4% formalin before anatomopathological study. These samples were used for further paraffin-embedding, slides, and HES histological staining. For transmission electron microscopy, the kidneys, livers, and tumors were cut into 1 mm^3^ fragments. Samples were fixed in pre-chilled 2% paraformaldehyde/2.5% glutaraldehyde in 0.1 M PBS pH 7.4 at 4°C for 3 h and processed as previously described [13].

### Biochemical analyses

Blood samples were collected from the tail vein of each rat under short isoflurane anesthesia before treatment and after 1, 2, 3, 6, 9, 12, 15, 18, 21, and 24 months: 11 measurements were obtained for each animal alive at 2 years. It was first demonstrated that anesthesia did not impact animal health. Two aliquots of plasma and serum were prepared and stored at −80°C. Then, 31 parameters were assessed (Table 1) according to standard methods including hematology and coagulation parameters, albumin, globulin, total protein concentration, creatinine, urea, calcium, sodium, potassium, chloride, inorganic phosphorus, triglycerides, glucose, total cholesterol, alanine aminotransferase, aspartate aminotransferase, gamma glutamyl-transferase (GT), estradiol, and testosterone. In addition, at months 12 and 24, the C-reactive protein was assayed. Urine samples were collected similarly 11 times, over 24 h in individual metabolic cages, and 16 parameters were quantified including creatinine, phosphorus, potassium, chloride, sodium, calcium, pH, and clearance. Liver samples taken at the end made it possible to perform assays of CYP1A1, 1A2, 3A4, 2C9 activities in S9 fractions, with glutathione S-transferase and gamma-GT.

### Statistical analysis

In this study, multivariate analyses were more appropriate than pairwise comparisons between groups because the parameters were very numerous, with samples of ten individuals. Kaplan-Meyer comparisons, for instance, were not used because these are better adapted to epidemiological studies. Differences in the numbers of mammary tumors were studied by a non-parametric multiple comparisons Kruskal-Wallis test, followed by a *post hoc* Dunn's test with the GraphPad Prism 5 software.

Biochemical data were treated by multivariate analysis with the SIMCA-P (V12) software (UMETRICS AB Umea, Sweden). The use of chemometrics tools, for example, principal component analysis (PCA), partial least squares to latent structures (PLS), and orthogonal PLS (OPLS), are robust methods for modeling, analyzing, and interpreting complex chemical and biological data. OPLS is a recent modification of the PLS method. PLS is a regression method used in order to find the relationship between two data tables referred to as *X* and *Y*. PLS regression [72] analysis consists in calculating by means of successive iterations, linear combinations of the measured *X*-variables (predictor variables). These linear combinations of *X*-variables give PLS components (score vectors *t*). A PLS component can be thought of as a new variable - a latent variable - reflecting the information in the original *X*-variables that is of relevance for modeling and predicting the response *Y*-variable by means of the maximization of the square of covariance (Max cov^2^(*X*,*Y*)). The number of components is determined by cross validation. SIMCA software uses the nonlinear iterative partial least squares algorithm (NIPALS) for the PLS regression. Orthogonal partial least squares discriminant analysis (OPLS-DA) was used in this study [73,74].

The purpose of discriminant analysis is to find a model that separates groups of observations on the basis of their *X* variables. The *X* matrix consists of the biochemical data. The *Y* matrix contains dummy variables which describe the group membership of each observation. Binary variables are used in order to encode a group identity. Discriminant analysis finds a discriminant plan in which the projected observations are well separated according to each group. The objective of OPLS is to divide the systematic variation in the *X*-block into two model parts, one linearly related to *Y* (in the case of a discriminant analysis, the group membership), and the other one unrelated (orthogonal) to *Y*. Components related to *Y* are called predictive, and those unrelated to *Y* are called orthogonal. This partitioning of the *X* data results in improved model transparency and interpretability [75]. Prior to analysis, variables were mean-centered and unit variance scaled.
